# Inhaled opioids for cancer pain relief: A narrative review

**DOI:** 10.1097/MD.0000000000028921

**Published:** 2022-06-24

**Authors:** Magdalena Osowicka, Piotr Janowiak, Agnieszka Gorzewska, Monika Lichodziejewska-Niemierko

**Affiliations:** a Department of Palliative Medicine, Medical University of Gdańsk, Gdańsk, Poland; b Department of Pulmonology, Medical University of Gdańsk, Gdańsk, Poland.

**Keywords:** aerosolized, analgesia, analgesic, inhalation, inhaled, nebulized, nebulized, pain

## Abstract

Despite the many new possibilities, cancer pain treatment is not always effective and often poses a challenge for practitioners. At the end-of-life care, both oral and subcutaneous drug delivery very often are not attainable. The increasing number of patients in terminal stage of chronic diseases forced us to look for the alternative ways of administration of pain treatment. In this context, the potentially rapid onset of action and ease of use make aerosolized drug delivery an attractive option in palliative care settings. The objective of this review was to identify literature on pain relief with inhaled opioids. The evidence suggests that nebulized opioids might be effective in the treatment of pain in various aetiologies; however, randomized controlled studies on nebulization therapy for cancer pain are lacking.

## 1 Introduction

Pain is one of the most important symptoms of advanced cancer.^[1]^ Insufficient therapy negatively affects quality of life, sleep, and physical function, and severely impairs normal daily activity.^[2,3]^ Therefore, effective pain management is a priority in palliative care. In moderate and severe pain, the treatment of choice is opioids.^[4]^ Although opioids are highly effective in relieving acute pain, their clinical utility can be limited by their side effects or the inability to administer them orally.^[5,6]^ The latter necessitates the search for alternative forms of drug administration. Commonly employed subcutaneous delivery might be complicated by local skin reactions and is contraindicated in patients with thrombocytopenia or blood coagulation disorders. In severe cases, subcutaneous injections may even result in phlegmon.^[7]^ In this context, the delivery of analgesics by nebulization might be an attractive alternative, considering that it is a preferred and effective route of opioid delivery in patients with episodic breathlessness.^[8,9]^ Thus, a literature review was performed to identify studies which assessed the effectiveness of nebulized opioids in the treatment of pain.

## 2 Methods

The MEDLINE database was searched using the following terms: inhaled, inhalation, nebulized, nebulized, aerosolized, pain, analgesia and analgesic. Studies on pain relief using nebulized opioids were included in this review. A single study on nebulized fentanyl in cancer patients was added to the review despite its absence in the MEDLINE database. Studies analysis is preceded by a short discussion on the significance of inhaled opioid pharmacokinetics. This is the literature review so the ethical approval was not necessary.

## 3 Results

The literature search provided 11 prospective studies on nebulized morphine and 11 on nebulized fentanyl. Tables 1 and 2 list the significant information on the aforementioned trials.

### 3.1 Inhaled opioids pharmacokinetics

The antinociceptive effect of opioids is related to their action on the μ, κ, and δ receptors located in the central nervous system and peripheral tissues.^[10]^ However, the bioavailability of nebulized opioids differs greatly depending on the type of nebulizer used.^[11]^ Systemic absorption of opioids, delivered by simple jet nebulizers, tends to have significant interindividual variability, as shown in pharmacokinetic studies of nebulized morphine^[12]^ and nebulized fentanyl.^[13]^ Furthermore, when using jet nebulizers, which deliver aerosols irrespective of patient respiration, one can expect significant variability in opioid deposition even between consecutive nebulization's in the same patient.^[14]^ This can be translated into an inconsistency of analgesia with a set dose of.^[12]^ It has been speculated that fentanyl, due to its lipophilic properties, is more readily absorbed through the respiratory tract than morphine.^[11]^ However, pharmacokinetic studies employing jet nebulizers contradict this assumption. The bioavailability of both nebulized morphine and fentanyl is low (5.5 ± 3.2%) and estimated at 10%.^[13,15]^

It is worth underlining that when opioids are delivered by modern nebulizers, pharmacokinetics changes considerably. AERx, a breath-actuated nebulizer calibrated for alveolar deposition, generates a bolus of small particle aerosol only when a proper inspiratory flow and volume are achieved.^[16]^ The bioavailability of morphine delivered by AERx can be as high as 66%, whereas that of fentanyl is 67%.^[11]^ Furthermore, the time courses of both morphine and fentanyl plasma concentrations in AERx pharmacokinetic studies were similar to their respective intravenous infusions, and so were their side effects profile.^[11]^ Pharmacokinetics close to intravenous and high bioavailability were also observed when fentanyl was delivered by low mass median aerodynamic diameter and high fine particle fraction metered-dose inhaler paired with SmartMist or Staccato delivery system.^[17,18]^ Another approach to opioid delivery was explored by Hung et al., who studied the pharmacokinetics of a mixture of free and liposome-encapsulated fentanyl which provided both rapid onset of absorption of the former and sustained absorption of the latter component.^[19]^

### 3.2 Nebulized morphine

Because of its potential ease of use in both prehospital and emergency department (ED) settings, there have been a few studies showing the usefulness of aerosolized morphine in reducing acute and traumatic pain. In their double-blind, randomized, controlled trial (RCT), Grissa et al compared 3 groups of 300 patients who were admitted to the ED due to a recent trauma.^[20]^ The primary end-points were a decrease in visual analogue scale (VAS) greater than or equal to 50% of its baseline value and pain resolution time. The drugs investigated were intravenous and nebulized morphine, the latter at 2 different doses (10 and 20 mg). Intravenous morphine was delivered in 2 mg boluses. Drug delivery was repeated every 5 minutes for the intravenous route and every 10 minutes for the nebulization route until the intended pain VAS drop. This study showed that nebulized morphine at both doses was non-inferior to intravenous morphine in terms of both primary endpoints. In addition, morphine delivered via nebulization produced fewer side effects, all of which were minor, with dizziness being the most frequent. The mean cumulative doses were higher for nebulized morphine that is, 11.4 mg in intravenous group, 21.2 mg in nebulized 10 mg morphine group and 36.5 mg in nebulized 20 mg group.^[20]^ Taking into consideration significant losses of drug aerosol when it is delivered via jet nebulizer, it is to no surprise – merely 12% of the set dose is deposited in the airways.^[21]^ In their double-blind RCT in 44 thoracic trauma patients, Fulda et al. showed similar results.^[22]^ Half of the study participants received nebulized morphine every 4 hours and continuous intravenous infusion (CIV) of 0.9% saline, whereas the other half received nebulized saline every 4 hours and CIV of morphine. Additional nebulization's and intravenous boluses were delivered on demand. If on-demand doses were dispensed frequently, both the nebulization dose and CIV rate were increased according to a predefined schedule. Mean post-treatment pain VAS scores between the 2 groups were similar that is 3.38 ± 1.8 for nebulized morphine versus 3.84 ± 2.7 for intravenous morphine. Average 4-hour nominal dose of morphine was, again, higher for nebulized morphine (11.96 mg vs 6.22 mg). Nonetheless, sedation scores were higher in the intravenous morphine group (modified Ramsay sedation scale [RSS]: 0.33 vs 0.56).^[22]^ No side effects of nebulized morphine, apart from minor sedation ranked 1 in RSS, were recorded in a prospective trial in thoracic trauma patients, conducted by Nejmi et al.^[23]^ The authors randomized 40 patients to receive either repeated doses of 8 mg nebulized morphine or epidural bupivacaine for 48 hours. The analgesic effect, where the primary endpoint was to obtain a pain VAS score of less than 4, was similar in both treatment arms, as well as sedation.^[23]^ In a prospective, uncontrolled study performed by Bounes et al, a single dose of 0.2 mg/kg nebulized morphine was unable to provide satisfactory analgesia, that is, a pain numeric rating scale (NRS) of 30/100 or lower.^[24]^ The study group consisted of 28 patients treated in the ED with severe acute pain that was 60 mm or higher. No side effects were noted.^[24]^ In contrast, when the same dose of nebulized morphine is allowed to be followed with repeated half-dose analgesia becomes effective, as shown in a convenience sample study by Lefevre et al.^[25]^ The effectiveness of nebulized morphine, fentanyl, and alfentanil, measured with verbal NRS, in 102 patients with acute pain who were admitted to the ED.^[25]^

Nebulized morphine treatment was also explored in an anesthetic setting. Chrubasik et al in their randomized controlled trial in 20 patients after abdominal surgery showed that continuous and on-demand nebulized morphine can be as effective as continuous and on-demand intravenous morphine with fewer side effects.^[26]^ However, the nebulized morphine group achieved verbally reported pain relief later (34 ± 9 vs 16 ± 3 minutes).^[26]^ On the other hand, a larger RCT among 52 abdominal surgery patients nebulized morphine analgesia was inferior to intravenous morphine.^[27]^ However, there were significant differences between the 2 groups. Surgeries in the nebulized morphine group lasted longer, upper abdominal surgeries were more frequent, and the initial pain VAS score was higher. Even though the nebulized morphine group received larger nominal dose of morphine (25.4 ± 1.1 mg vs 23.4 ± 0.8 mg), serum morphine concentrations were higher in the intravenous group. It comes as no surprise that side effects, including sedation, were more frequent in the latter. However, because of the low sedation rate, postoperative restlessness was more frequent in the nebulized morphine group.^[27]^ In the other 2 RCTs, in patients after cardiac surgery, Chrubasik et al showed that nebulized morphine can be effectively used as an analgesic.^[27]^ In the first study, a pilot study of 40 cardiac surgery patients showed that nebulized morphine was clearly superior to nebulized saline, whereas in a subsequent study, in 30 patients, nebulized morphine provided a similar analgesic effect as intravenous morphine but with less side-effects.^[27]^ In both studies, analgesia was assessed using additional meperidine consumption. However, 7 patients in the nebulized morphine group required additional diazepam because of restlessness.^[27]^ Other authors have shown the possible efficacy of nebulized morphine in pre-emptive analgesia before septoplasty or septorhinoplasty.^[28]^ In their double-blind RCT, Onal et al randomized 80 patients to either nebulized 65 μg/kg morphine or nebulized saline.^[28]^ Nebulization, delivered by a jet nebulizer, was performed 10 minutes prior to induction. It was noted that the first analgesia requirement emerged significantly later in the nebulized morphine group that is231.72 versus 48.75 minutes. No significant difference was detected between the frequency of postoperative nausea or vomiting.^[28]^

As mentioned earlier, nebulized morphine pharmacokinetics depend significantly on the type of nebulizer used. The studies mentioned above exclusively employed jet nebulizers which produce low serum morphine concentrations. However, Thipphawong et al in their double-blind RCT of nebulized morphine in bunionectomy patients used an AERx nebulizer, designed to deposit most of the drug aerosol in the alveoli.^[29]^ In this study, 89 patients were divided into 4 treatment arms: a) nebulization of 1 unit dose containing 2.2 mg of morphine, taking bioavailability into consideration, 1.4 mg would be delivered systemically, b) nebulization of 3 analogous unit doses, c) 4 mg of intravenous morphine, and d) placebo. Rescue medication, that is, 2 mg of intravenous morphine, was available in all treatment arms. As expected, 4 mg of intravenous morphine provided a similar analgesic effect, including onset time, as nebulization of 3 unit doses.^[29]^

In the only study of nebulized morphine in palliative setting Majidinejad et al compared nebulized morphine with oral methadone and transdermal fentanyl in a RCT in 90 end-stage cancer patients.^[30]^ Patients were hospitalized for 3 days, and pain severity was measured twice daily using the VAS. The nebulized morphine group received it at a dose of 20 mg, repeated every 10 minutes, with a maximum of 3 doses. After 3 days of treatment, nebulized morphine provided analgesia similar to oral methadone, delivered at a maximum daily dose of 45 mg, and 0.6 mg of transdermal fentanyl. The only side effect in the nebulized morphine group was dizziness, which was reported in 2 out of 30 patients. Nonetheless, according to the authors, the study was underpowered to compare the side effects profile.^[30]^

As shown above, nebulized morphine frequently provided analgesia similar to its intravenous route, but with limited side effects. This could be explained by the different metabolite profiles. Krajnik et al showed that when morphine is delivered via nebulization, morphine-6-glucuronide (M6G) might be synthesized in almost the same amount as morphine-3-glucoronide (M3G), whereas morphine delivered by any other route is mostly metabolized to M3G with M3G to M6G ratios ranging from 3.12 to 11.00.^[31–33]^ The increase in M6G generation might be a result of UGT2B7 glucuronidase activity, which is capable of almost equal morphine glucuronidation to M3G and M6G, and was detected in human lung specimens.^[34]^ Furthermore, M6G is the metabolite which is mainly responsible for morphine's analgesic effect, while M3G is mostly associated with its side effects.^[35]^

### 3.3 Nebulized fentanyl

Fentanyl, due to its lipophilicity and fast absorption rate, is already used in the treatment of acute or breakthrough pain in the home setting when delivered intranasally or transmucosally.^[5]^ However, intranasal or transmucosal absorption can be limited by mucosal inflammation, increased secretion, and excessive drying; the latter is often induced by oncological treatment.^[5,36]^ Dry mouth, secondary to the dysfunction of the salivary glands, may affect up to 80% of patients with advanced neoplastic disease, hindering the dissolution of buccal tablets, increasing discomfort, and reducing the effectiveness of therapy.^[36]^

Another potential route of fentanyl delivery is nebulization. Nebulized fentanyl was first studied as an analgesic in postoperative patients. Worsley et al performed a small, single-blind, randomized, controlled study of 20 patients after a variety of elective surgical procedures showed that a single dose of nebulized 300 μg fentanyl significantly reduced pain VAS scores and prolonged time to alternative analgesia in 7 patients, when compared with 13 patients who received a single dose of saline or nebulized 100 μg fentanyl.^[13]^ Singh et al compared nebulized fentanyl in two doses: 3 and 4 μg/kg with 2 μg/kg intravenous fentanyl in a double-blind, randomized, controlled study in 90 patients after lower abdominal surgery, equally divided into 3 groups.^[37]^ The authors showed that higher doses of nebulized fentanyl produced analgesia, as measured by VAS, similar to the intravenous route, yet delayed (10 minutes vs 5 minutes) and prolonged (90 minutes vs 30 minutes). The side effects were comparable between groups. However, sedation, as measured by RSS, was always less prominent with nebulized fentanyl which can be explained by the slower rise of nebulized fentanyl peak levels.^[37]^ Similar findings were reported by El-Hamid et al, who replicated the study by Singh et al.^[38]^ In a double-blind randomized controlled trial among 87 patients after unilateral arthroscopic anterior cruciate ligament reconstruction surgery authors showed that 4 μg/kg of nebulized fentanyl provided longer (80.5 ± 11.52 minutes vs 74.7 ± 9.81 minutes), yet slightly delayed analgesia (5.13 ± 1.16 minutes vs 4.55 ± 1.18 minutes) with less sedation when compared with 2 μg/kg intravenous fentanyl.^[38]^ Even longer postoperative analgesia was reported by Clark et al, who tested a patient-titrated nebulized mixture of free and liposome-encapsulated fentanyl in 19 postsurgery patients.^[39]^ Eighteen of them achieved effective analgesia at a median time of 17 min after nebulization which lasted for a mean 3.7 hours.^[39]^ Postoperative fentanyl analgesia was also explored by Higgins et al, who compared the effects of 3 concentrations of nebulized fentanyl on pain VAS in a double-blind study among 30 patients after both laparoscopic and non-laparoscopic procedures.^[40]^ Patients who received 960 μg had better pain control (mean VAS change: −3.4 cm) than those who received 480 μg (mean VAS change: −0.8 cm) and 190 μg (mean VAS change: −1.7 cm). However, patients rated their pain only twice, before and 5 minutes after nebulization, and received intravenous morphine if pain control after 5 minutes was not satisfactory. Most patients who received lower doses of nebulized fentanyl (480 μg and 190 μg) received morphine 5 minutes after nebulization, whereas Singh showed that nebulized fentanyl analgesia sets in on average 10 minutes after nebulization.^[37,40]^ Higgins et al also underlined the potential drawbacks of delivering nebulized fentanyl in the postoperative setting, that is, the requirement of constant supervision and dependence of analgesia on variable aerosol deposition if nebulization is provided via standard jet nebulizers.^[40]^

Further research showed that inhaled fentanyl might be an effective alternative for acute pain in the ED setting, especially when intravenous access is not feasible.^[41]^ In their randomized, double-blind, controlled study, Deaton et al. compared the effectiveness of nebulized fentanyl (2 μg/kg) with 0.1 mg/kg intravenous morphine in alleviating acute, non-injury, and abdominal pain in 32 patients who were admitted to the ED.^[41]^ It should be emphasized that fentanyl in this study was nebulized with a breath-actuated jet nebulizer, without virtually any loss of nebulized drug into the environment. Patients treated with nebulized fentanyl had both clinically and statistically significant pain relief (highest mean VAS change, −37.48 mm), which was significantly larger than that in the intravenous morphine group (highest mean VAS change: −16.63 mm). Furthermore, nebulized fentanyl analgesia was more rapid and lasted longer than the 40-minute study interval. No side effects were noted in the nebulized fentanyl group compared to 7 out of 16 patients treated with intravenous morphine who required antiemetics. Treatment of abdominal pain with nebulized fentanyl was also explored by Bartfield et al, who compared its effectiveness with intravenous fentanyl in a double-blind randomized controlled study of 50 patients.^[42]^ Pain VAS scores were measured at baseline and at 15 and 30 minutes after the study medications. 1.5 μg/kg of nebulized fentanyl, which was delivered with a breath-actuated jet nebulizer, was shown to be non-inferior to 1.5 μg/kg of intravenous fentanyl in terms of pain VAS reduction (−16 mm vs −25 mm) and the need for rescue medication at 30 minutes post treatment delivery. As in earlier studies, nebulized fentanyl delivered analgesia with a certain delay, that is, there was a significant difference in pain VAS at 15 minutes between nebulized and intravenous fentanyl. No adverse events were recorded in both groups.^[42]^ In another double-blind, randomized, controlled trial, Farahmand et al compared the effectiveness of intravenous morphine (4 μg/kg) and 0.1 mg/kg of intravenous morphine in 90 patients with acute limb pain.^[43]^ Pain NRS was assessed at 5, 10, 15, 30, 45, and 60 minutes after drug administration. Fifteen minutes of nebulized fentanyl was shown to provide slightly stronger analgesia than intravenous morphine, although the difference was not clinically significant (highest mean NRS change: −5.2 vs −4.6). No differences in delay in analgesia and rescue dose usage were detected between the 2 groups. No adverse effects were reported in nebulized fentanyl group.^[43]^

Delivering analgesia via aerosol might be especially convenient in children in whom insertion of an intravenous cannula or intramuscular injection can be a source of significant distress. Two non-blinded, randomized controlled studies have explored this possibility. In their study in 73 children with suspected limb fractures Furyk et al showed that analgesia with 4 μg/kg of nebulized fentanyl, delivered by standard jet nebulizer, is non-inferior to 1 provided with 0.1 mg/kg of intravenous morphine that is, mean Wong and Baker faces pain scale change with nebulized fentanyl was equal −3.60 whereas with intravenous morphine: −3.00.^[44]^ In a study by Miner et al on 41 children admitted to the ED with significant pain of various aetiology 3.0 μg/kg of nebulized fentanyl (n = 27) provided similar analgesia to 1.5 μg/kg of intravenous fentanyl (n = 14).^[45]^ However, 4 patients in the nebulized fentanyl group, all younger than 3 years, had significant difficulty in triggering the breath-actuated nebulizer employed in this study. Analgesia was assessed using the physician VAS score, patient VAS score (6/27 and 5/14 children), and Children's Hospital of Eastern Ontario Pain Scale score (21/27 and 9/14 children).^[45]^

In a single identified trial of nebulized fentanyl for cancer pain, Boyle et al employed the AERx nebulization system.^[46]^ In this open-label, single-visit, multiple-dose study, 20 patients achieved satisfactory breakthrough pain relief within a limit of up to 3 doses of 200 μg nebulized fentanyl. No serious adverse events were observed, and few patients reported dizziness, emesis, nausea and light-headedness.^[46]^

## 4 Discussion

The limited published data suggest that delivering morphine or fentanyl via nebulization might provide effective analgesia which is non-inferior to its parenteral counterparts. As shown in the studies discussed above, no serious side effects were observed, and nebulization was not complicated by respiratory symptoms. Some studies have suggested that delivering opioids by nebulization might result in a lower side effect rate, especially with regard to sedation. The claim of treatment safety is also supported by studies on treating dyspnea with nebulized opioids.^[47]^ Despite the constant development of aerosol therapy, research, especially employing modern, efficient inhalers, is lacking. Two studies have explored the possibility of treating cancer pain with nebulized opioids, while both showed positive results.^[30,46]^ The authors’ view that the potential advantages of treating pain with nebulized opioids, such as ease of use by both patients and guardians or the ability to deliver medication when other routes are not feasible, merit further research.
